# Authors reply to the ‘Response to “Similarities in clinical course and outcome between juvenile idiopathic arthritis (JIA)-associated and ANA-positive idiopathic anterior uveitis: data from a population-based nationwide study in Germany”’

**DOI:** 10.1186/s13075-023-03017-7

**Published:** 2023-03-29

**Authors:** Arnd Heiligenhaus, Jens Klotsche, Kirsten Minden

**Affiliations:** 1 Department of Ophthalmology at St. Franziskus Hospital, Muenster, Hohenzollernring 74, 48145 Muenster, Germany; 2 University of Duisburg-Essen, Duisburg, Germany; 3 German Rheumatism Research Center, a Leibniz Institute, Berlin, Germany; 4 Epidemiology and Health Economics, Charité – University Medicine Berlin, Institute for Social Medicine, Berlin, Germany; 5 Department of Rheumatology and Clinical Immunology, Charité - University Medicine Berlin, Berlin, Germany

First, the data presented in our population-based nationwide National Paediatric Rheumatological Database including detailed uveitis documentation in Germany compared the 2-year follow-up data of ANA-positive patients with idiopathic anterior uveitis, patients with initial uveitis diagnosis after JIA onset, and JIA patients with initial uveitis diagnosis before arthritis onset. Herein, uveitis course, anti-inflammatory treatment, and response to corticosteroids and disease-modifying anti-rheumatic drugs (DMARDs) were compared [1]. As the follow-up rate declined with longer follow-up (FU), our analysis was restricted to 2-year FU.

Second, remission was defined in accordance to Multinational Interdisciplinary Working Group for Uveitis in Childhood [2]. Only patients with uveitis inactivity for the defined time-point were encountered. Hereby, remission has been defined as uveitis inactivity at 2-year FU under ophthalmic care, while the exact number of previous uveitis flares could not be provided within our study.

Third, patients with insidious onset of disease being ANA positive are at particular risk of developing JIA, as uveitis onset may occur before arthritis onset. The issue of probable vision-threatening course of anterior uveitis without systemic disease (e.g., JIA) has been generally underappreciated, while it was highlighted by Holland et al. [3]. Our assumption herein is also in line with other recent papers and guidelines thereby stressing that JIA-associated uveitis and ANA pos. anterior uveitis should be treated in the same way [4]. The data within our study were prospective, while the papers mentioned in the letter were review papers. While the authors of the letter described the importance of recognizing and preventing blindness from JIA-associated uveitis, we intended to highlight the high burden of disease also in the particular group of children with anterior uveitis being ANA pos. but without JIA, with our observation also requiring close monitoring and adequate disease management, including DMARD use. This assumption is supported by data from a multicenter, double-blind, randomized, placebo-controlled trial [5]. The study led to the approval of adalimumab for the treatment of chronic, non-infectious anterior uveitis in pediatric patients from 2 years of age who have had an inadequate response to conventional therapy, either with or without associated JIA.

## References

[1] Heiligenhaus A, Klotsche J, Niewerth M, Horneff G, Ganser G, Haas JP, Minden K (2020). Similarities in clinical course and outcome between juvenile idiopathic arthritis (JIA)-associated and ANA-positive idiopathic anterior uveitis: data from a population-based nationwide study in Germany. Arthritis Res Ther.

[2] Foeldvari I, Klotsche J, Simonini G, Edelsten C, Angeles-Han ST, Bangsgaard R (2019). Proposal for a definition for response to treatment, inactive disease and damage for JIA associated uveitis based on the validation of uveitis related JIA outcome measures from the Multinational Interdisciplinary Working Group for Uveitis in Childhood (MIWGUC). Pediatr Rheumatol Online J.

[3] Holland GN, Denove CS, Yu F (2009). Chronic anterior uveitis in children: clinical characteristics and complications. Am J Ophthalmol.

[4] Angeles-Han ST, Lo MS, Henderson LA, Lerman MA, Abramson L, Cooper AM (2019). Childhood Arthritis and Rheumatology Research Alliance consensus treatment plans for juvenile idiopathic arthritis-associated and idiopathic chronic anterior uveitis. Arthritis Care Res (Hoboken).

[5] Ramanan AV, Dick AD, Jones AP, McKay A, Williamson PR, Compeyrot-Lacassagne S (2017). Adalimumab plus methotrexate for uveitis in juvenile idiopathic arthritis. N Engl J Med.

